# Eicosapentaenoic acid preserves diaphragm force generation following endotoxin administration

**DOI:** 10.1186/cc8913

**Published:** 2010-03-16

**Authors:** Gerald S Supinski, Jonas Vanags, Leigh Ann Callahan

**Affiliations:** 1 Division of Pulmonary, Critical Care and Sleep Medicine, University of Kentucky, 740 South Limestone, L-543, Lexington, KY 40536-0284, USA

## Abstract

**Introduction:**

Infections produce severe respiratory muscle weakness, which contributes to the development of respiratory failure. An effective, safe therapy to prevent respiratory muscle dysfunction in infected patients has not been defined. This study examined the effect of eicosapentaenoic acid (EPA), an immunomodulator that can be safely administered to patients, on diaphragm force generation following endotoxin administration.

**Methods:**

Rats were administered the following (n = 5/group): (a) saline, (b) endotoxin, 12 mg/kg IP, (c) endotoxin + EPA (1.0 g/kg/d), and (d) EPA alone. Diaphragms were removed and measurements made of the diaphragm force-frequency curve, calpain activation, caspase activation, and protein carbonyl levels.

**Results:**

Endotoxin elicited large reductions in diaphragm specific force generation (*P *< 0.001), and increased diaphragm caspase activation (*P *< 0.01), calpain activation (*P *< 0.001) and protein carbonyl levels (*P *< 0.01). EPA administration attenuated endotoxin-induced reductions in diaphragm specific force, with maximum specific force levels of 27 ± 1, 14 ± 1, 23 ± 1, and 24 ± 1 N/cm^2^, respectively, for control, endotoxin, endotoxin + EPA, and EPA treated groups (*P *< 0.001). EPA did not prevent endotoxin induced caspase activation or protein carbonyl formation but significantly reduced calpain activation (*P *< 0.02).

**Conclusions:**

These data indicate that endotoxin-induced reductions in diaphragm specific force generation can be partially prevented by administration of EPA, a nontoxic biopharmaceutical that can be safely given to patients. We speculate that it may be possible to reduce infection-induced skeletal muscle weakness in critically ill patients by administration of EPA.

## Introduction

Infections produce severe respiratory muscle weakness, which contributes to the development of respiratory failure [1,2]. An effective, safe therapy to prevent respiratory muscle dysfunction in infected patients has not been defined. One approach to identifying a potential therapeutic agent would be to evaluate the effects of agents that can block cellular pathways known to be pathophysiologically linked to the development of infection induced muscle dysfunction [3]. One such pathway is the caspase proteolytic pathway, and infections have been shown to activate the extrinsic caspase pathway, increasing skeletal muscle caspase 3 activity [4]. In addition, infections stimulate intramuscular generation of high levels of free radicals, activate the skeletal muscle proteasomal proteolytic pathway, and trigger the skeletal muscle calpain proteolytic system [5-7]. Each of these pathways, moreover, is theoretically capable of interacting with skeletal muscle contractile proteins and thereby reducing force generation [8-10].

One pharmacologic agent, eicosapentaenoic acid (EPA), inhibits caspase activation, reduces proteasomal pathway activation, and is a free radical scavenger. Specfically, EPA has been shown to inhibit activation of both the intrinsic (that is, mitochondrial) and extrinsic (death receptor related) caspase activation pathways [11]. This agent has also been reported to be a weak free radical scavenger and to have nonspecific antioxidant effects [12]. More recently, this agent has been reported to act as a proteasome inhibitor, blocking proteolysis and muscle wasting in patients with pancreatic carcinoma [13], reducing proteasomal activation in an animal model of sepsis [14], and reducing the resting level of cytokines [15]. Moreover, EPA is well tolerated and has been safely administered to patients without reports of toxicity in a large number of studies [12,13,16]. Based on these past reports, it is reasonable to postulate that it may be rational to utilize EPA to prevent muscle weakness in infected patients. No previous study, however, has examined the effect of EPA on skeletal muscle force generating capacity, and it is not known if this agent can prevent skeletal muscle weakness in either patients or animal models of infection.

The purpose of the present experiment, therefore, was to test the hypothesis that administration of EPA would attenuate the effects of endotoxin (LPS) induced sepsis on diaphragm force generating capacity. Studies were conducted in rats and comparison made of diaphragm force generation, protein content, and muscle mass between groups of control animals, animals treated with endotoxin, animals given both endotoxin and EPA, and animals given EPA alone. We also sought to determine if EPA administration attenuates the activation of one or more of the downstream mechanisms by which endotoxin administration alters skeletal muscle force generation. To do so, we measured indices of caspase 3 activation, free radical formation, and calpain activity for diaphragm samples taken from the four animal groups studied in this experiment.

## Materials and methods

### Protocol

Experiments were performed using rats 250 to 350 g in weight. Approval for this work was granted by the Institutional Animal Care and Use Committee. Animals were given food and water ad lib and housed in university facilities. Saline (60 mg/kg/d) was administered subcutaneously to maintain fluid volume status. Animals were sedated with pentobarbital (50 mg/kg intraperitoneally) before euthanasia.

Studies were performed on groups of rats (n = 5/group) given: (a) saline, 0.3 ml IP and a 0.5 ml olive oil gavage/d, (b) endotoxin, 12 mg/kg of *E. coli *lipopolysaccharide administered in 0.3 ml saline, Sigma Chemicals, St. Louis, MO, USA IP, and a 0.5 ml olive oil gavage/d, (c) endotoxin and EPA (1.0 g/kg/d administered in a 0.5 ml olive oil gavage), and (d) saline, 0.3 ml IP, and EPA (1.0 g/kg/d administered in a 0.5 ml olive oil gavage). EPA was administered in this dosage and given in an olive oil gavage based on previous work using this agent [17,18]. Animals were killed at 48 hours because previous work in our laboratory found that diaphragm specific force levels fall to a nadir by that time after endotoxin administration [19,20]. At the time of sacrifice, diaphragms were removed and the following were measured: (a) diaphragm weight, (b) total diaphragm protein levels, (c) the diaphragm force-frequency relationship, (d) caspase 3 activity using a fluorogenic assay, (e) diaphragm carbonyl content, an index of free radical generation, (f) calpain activity using a fluorogenic assay, and (g) procalpain I and active calpain I levels using Western blotting. No animals died before tissues could be harvested.

### Determination of diaphragm specific force generation

Diaphragm force generation was assessed as we have previously reported [21]. In brief, after diaphragms were excised and placed in a dissecting dish, muscle strips were dissected from the left mid-costal portion. Strips were then mounted vertically in water jacketed glass organ baths containing Krebs-Henselheit solution (25°C, curare 50 mg/l, pH 7.40, NaCl 135 mM, KCl 5 mM, dextrose 11.1 mM, CaCl_2 _2.5 mM, MgSO_4 _1 mM, NaHCO_3 _14.9 mM, NaHPO_4 _1 mM, insulin 50 units/L, 95% O_2_/5% CO_2_). One end of each strip was tied to the base of the organ bath and the other end to a SI force transducer (Scientific Instruments, Heidelberg, Germany). Platinum mesh field electrodes were used to deliver supramaximal currents using a biphasic constant current amplifier driven by a Grass S48 stimulator (Grass, West Warwick, RI, USA). After a 15 minute equilibration period, muscle strip length was adjusted to L_o_, that is, the length at which strip force generation in response to single stimuli was maximal. Strips were then sequentially stimulated with trains of 1, 10, 20, 50, and 80 Hz. stimuli (train duration 800 msec, 30 sec between adjacent trains) and force recorded with a Gould 2600 strip chart recorder (Gould, Cleveland, Ohio, USA). A cross sectional area was calculated as muscle strip weight divided by muscle density (1.06) and muscle length [22]. Specific muscle force was calculated as raw force divided by cross sectional area.

### Assessment of diaphragm calpain and caspase 3 activity

For calpain and caspase activity assays, diaphragm samples were first homogenized in a 1 g/10 ml ratio in assay buffer (50 mM HEPES, 100 mM NHCl, 0.1% CHAPS, 10 mM DTT, I mM EDTA, 10% glycerol, pH 7.4) using a polytron while on ice. Samples were centrifuged at 10,000 G for 10 minutes at 4°C, the supernatant removed and assayed for protein content. Diaphragm homogenates (100 micrograms of supernatant protein) were added to assay buffer and either a calpain-specific fluorogenic substrate (Suc-LLVY-AMC, that is, Succinyl-Leu-Leu-Val-Tyr-7-amido-4-methyl-coumarin) or a caspase 3 specific substrate (Ac-DEVD-AMC, that is, N-acetyl-Asp-Glu-Val-Asp-7-amino-4-methylcoumarin). Duplicate determinations were made for each sample using a mixture of diaphragm homogenate, assay buffer, fluorogenic substrate and either a highly specific calpain inhibitor (0.1 mg/ml calpain inhibitor III, carbobenzoxy-valinyl-phenylalaninal) or a caspase 3 inhibitor (20 nM DEVD-CHO, that is, N-acetyl-Asp-Glu-Val-Asp-aldehyde). Immediately after the addition of the fluorogenic substrate, a baseline measurement of AMC (7-Amino-4-methylcoumarin) was performed using a Molecular Devices spectrofluorophotometer (Molecular Devices, Silicon Valley, CA, USA) (excitation frequency of 360 nm and emission frequency of 460 nm). This measurement was repeated after 0.5 hour of incubation at 30°C for caspase assays and after 0.5 hour of incubation at 25°C for calpain assays. AMC standards were used to create a calibration curve and activity was quantified as nmoles of AMC generated/min/mg of tissue homogenate. The difference between AMC generation from incubation of homogenates with Suc-LLVY-AMC in the presence and absence of calpain inhibitor III was used as an index of calpain activity; note that calpain inhibitor III inhibits calpain activity but does not inhibit proteasomal activity. Similarly, the difference between AMC generation from incubation of homogenates with Ac-DEVD-AMC in the presence and absence of DEVD-CHO was used as an index of caspase 3 activity. Note that DEVD-CHO does not inhibit calpain, caspase 8 or caspase 9 but specifically inhibits caspase 3.

### Western blots and oxyblot protein modification determinations

SDS-PAGE protein gels were run for Western blot analysis of specific proteins (that is, calpain I protein, Oxyblot protein carbonyl sidegroup formation). The purpose of assessing calpain I protein was to detect the presence of the 78 kDa calpain I cleavage product. This cleavage product is known to be formed autocatalytically when calpain I is activated. Arguably, this is a superior means of determining if calpain I is active in intact tissues than many traditional indices. For example, some techniques to assess calpain activation employ initial isolation of calpain I, calpain II, and/or calpastatin from tissues and subsequent assessment of calpain or calpastatin activity of the isolated proteins (for example, zymography), but this approach assesses the in vitro activity of these enzymes or their inhibitor (calpastatin) and does not determine the activity of these enzymes under *in vivo *conditions. In contrast, the presence of the 78 kDa calpain I cleavage product in diaphragm homogenates can only occur from *in vivo *calpain I activation in the diaphragm.

The specifics for performing Oxyblot and calpain I blots are as follows. Prior to SDS-PAGE gel analysis, samples (10 μg/lane) used for Oxyblot determination were prepared as per the manufacturer's instructions (Oxyblot Kit, Chemicon, Temecula, CA, USA). Nonderived protein samples for calpain I determinations (100 μg/lane) were diluted with an equal volume of a loading buffer (126 mM Tris-HCL, 20% glycerol, 4% SDS, 1.0% 2-mercaptoethanol, 0.005% bromphenyl blue, pH 6.8). Samples were then loaded onto 12% Tris Glycine polyacrylamide gels and proteins separated by electrophoresis (Novex Minicell II; Invitrogen, Carlsbad, Ca. USA). Standard molecular weight markers were also loaded onto gels to permit approximation of protein band molecular weights. After electrophoresis, proteins were transferred to polyvinylidene fluoride membranes and incubated over night at 4°C with antibodies to targeted proteins (anti-calpain I, Chemicon, and anti-DNP antibody for Oxyblot determination, Chemicon). Membranes were then incubated with horse radish peroxidase (HRP) conjugated secondary antibodies and antibody binding was detected using enhanced chemiluminescence (NEN Life Science Products, Boston, MA, USA). Gel densitometry was performed using a Microtek scanner (Carson, CA, USA) and UN-SCAN-IT software (Silk Scientific, Orem, UT, USA). Since calpain I cleavage is an index of in vivo calpain I activation, calpain densitometry is reported as the ratio of cleaved calpain I to intact calpain I protein levels. This normalization procedure also corrects for lane to lane differences in protein loading; to further assess protein loading, parallel gels were run probing for α-tubulin.

### Statistics

ANOVA was used for comparison of parameters across experimental groups. Tukeys test was used to determine differences between individual groups following ANOVA. A *P*-value of less than 0.05 was taken as indicating statistical significance. Data are presented as mean ± SEM (standard error of mean).

## Results

### Effects of endotoxin and EPA on the diaphragm force-frequency relationship

**Figure 1:**
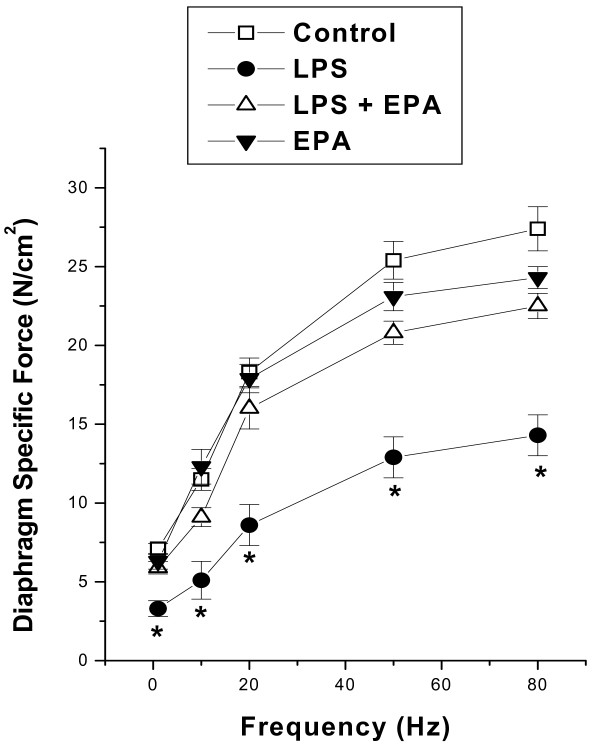
**Diaphragm force-frequency curves**. Force generation was significantly lower at stimulation frequencies from 1 to 80 Hz for diaphragms from LPS treated animals (filled circles) than for control animals (open squares) (*P *< 0.001). Diaphragms from animals given both EPA (eicosapentanoic acid) and LPS generated forces significantly higher than diaphragms from animals given LPS alone for stimulation frequencies of 1, 20, 50 and 80 Hz (*P *< 0.01 for each comparison). Forces generated by the EPA+LPS group were similar to controls for 1, 10 and 20 Hz but were lower than controls for 50 and 80 Hz (*P *< 0.05). Force generation for muscles taken from animals given EPA alone was similar to levels for control animals. * indicates a significant statistical difference between endotoxin and all other groups.

Endotoxin (LPS) administration produced a large reduction in diaphragm specific force generation, that is, the force generated per unit cross sectional area (Figure 1). LPS induced reductions in diaphragm force generation were evident for all stimulation frequencies tested (1 to 80 Hz), with maximum force reduced by 48% when compared to values for control experiments (*P *< 0.001 for comparison of 80 Hz force for control and LPS treated groups). More importantly, EPA administration substantially attenuated this LPS induced reduction in diaphragm force, with the specific force-frequency curve for the LPS + EPA group lying above the curve for the LPS treated group (*P *< 0.01 for comparison of specific force between LPS and LPS + EPA treated groups at stimulation frequencies 1, 20, 50, and 80 Hz). The EPA + LPS group was similar to control levels for stimulation frequencies 1 to 20 Hz but remained lower than control levels for 50 and 80 Hz force measurements (*P *< 0.05 for these latter comparisons).

### Effects on diaphragm mass (weight) and protein content

At 48 hours following endotoxin (LPS) administration, diaphragm weight (mass) and protein content remained at levels similar to those measured on diaphragms from control animals. Specifically, diaphragm weight averaged 1.73 ± 0.05, 1.69 ± 0.04, 1.58 ± 0.09, and 1.90 ± 0.10 g/initial body weight-g, respectively for control, LPS, LPS + EPA and EPA treated groups (NS). In addition, diaphragm protein levels were similar across the four groups, averaging 0.36 ± 0.02, 0.32 ± 0.03, 0.31 ± 0.03, and 0.37 ± 0.02 g/initial body weight-g, respectively, for control, LPS, LPS + EPA, and EPA alone treated groups (NS).

### Effects on activation of caspase 3, protein carbonyl formation and calpain activation

**Figure 2:**
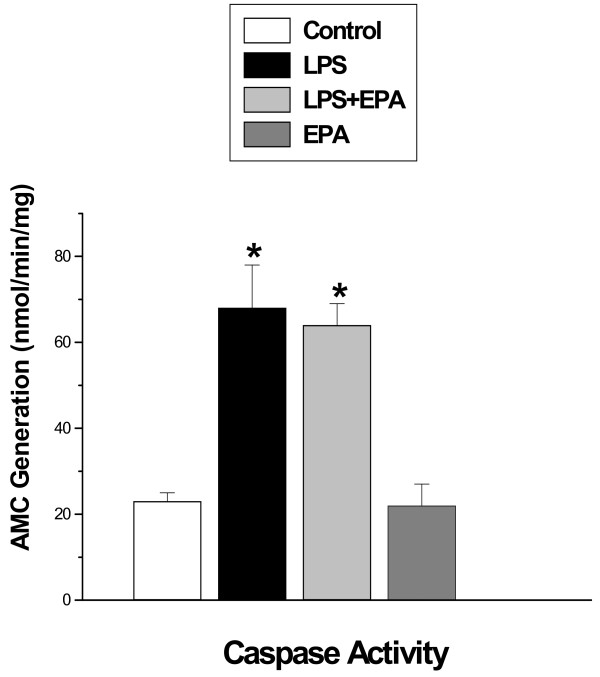
**Diaphragm caspase 3 activity**. Diaphragm caspase 3 activity was significantly increased for samples taken from animals after LPS treatment (filled bar) as compared to controls (open bar) (*P *< 0.01). EPA administration did not prevent this increase in diaphragm caspase 3 activity, with caspase 3 activity higher for the EPA plus LPS group (light grey bar) as compared to controls (*P *< 0.01). This parameter was similar in control and the EPA alone group (dark grey bar) and the EPA alone group was different from the LPS alone group (*P *< 0.01). * indicates a significant statistical difference compared to control and EPA alone groups.

**Figure 3:**
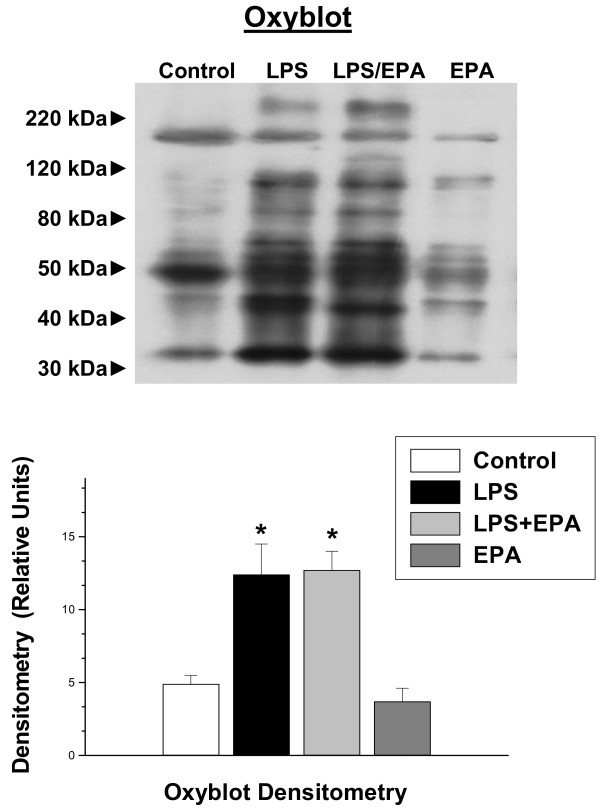
**Oxyblot determination**. Diaphragm protein carbonyl content, as assessed by the oxyblot technique, is shown for representative diaphragm samples in the top blots while group mean densitometry data is presented in the bottom graph. Protein carbonyl levels were increased for diaphragm samples taken from animals after LPS treatment as compared to control animals (*P *< 0.01). EPA administration did not reduce carbonyl levels significantly in LPS treated animals. Carbonyl levels for animals given EPA alone were similar to levels in saline treated controls. * indicates a significant statistical difference compared to control and the EPA alone groups.

Previous work indicates that LPS reduces diaphragm specific force generation by several mechanisms, including via the effects of diaphragm caspase activation, free radical induced alterations in protein function and through the actions of activated calpain. We therefore sought to determine if EPA administration blocked activation of one or more of these LPS-induced processes. We found that LPS administration induced an increase in diaphragm caspase 3 activity, as judged by the ability of diaphragm homogenates to cleave a caspase 3 specific fluorogenic substrate (*P *< 0.01, Figure 2). Administration of EPA, however, had no effect on LPS-induced diaphragm caspase 3 activation, which remained significantly higher for the LPS + EPA group compared to control (*P *< 0.01). We also found that LPS administration significantly increased diaphragm protein carbonyl content (see Figure 3, *P *< 0.01 for carbonyl densitometry levels between control and LPS treated groups). EPA administration also failed to attenuate protein carbonyl formation, with protein carbonyl densitometry for the LPS + EPA group similar to that of the LPS alone group and higher (*P *< 0.05) than values for controls.

**Figure 4:**
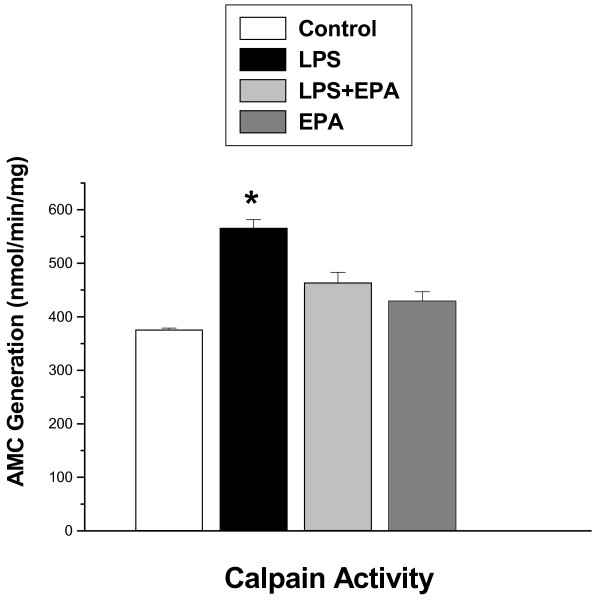
**Diaphragm calpain activity**. Diaphragm calpain activity was significantly increased for samples taken from animals after LPS treatment (filled bar) as compared to controls (open bar) (*P *< 0.001). EPA administration reduced this increase in diaphragm calpain activity, with calpain activity lower for the EPA plus LPS group (light grey bar) as compared to LPS treated animals (*P *< 0.02). Diaphragm calpain activity for samples from animals given EPA alone (dark grey bar) was similar to levels for the control group. * indicates a significant statistical difference from the other groups.

**Figure 5:**
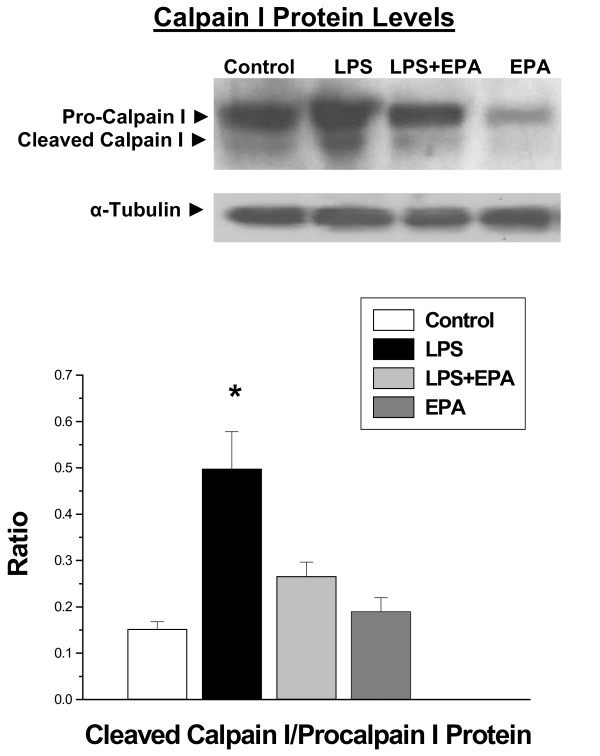
**Calpain I protein levels for diaphragm samples**. Western blots stained for calpain I protein, with top panels presenting blots for representative samples and the bottom graph providing group mean densitometry data for cleaved calpain I/intact calpain I protein levels. Blots were reprobed with alpha tubulin to verify equal loading of lanes. Levels of cleaved calpain I (78 kDa protein)/total calpain I were higher for samples after LPS treatment (filled bars) than for samples from control animals (open bars), animals given both LPS and EPA (light grey bars) or animals given EPA alone (dark grey bars)(*P *< 0.02). * indicates a significant statistical difference from the other groups.

In contrast, EPA largely prevented LPS induced calpain activation (Figures 4 and 5). In keeping with previous reports, we found that LPS significantly increased diaphragm calpain activation as measured using a fluorogenic substrate based assay (*P *< 0.001, Figure 4) and by measuring diaphragm levels of cleaved calpain I protein (that is, active, cleaved 78 kDa calpain I protein, *P *< 0.01, Figure 5). EPA blocked both of these increases, with calpain activity levels for the LPS + EPA group significantly lower than levels for the LPS alone group (*P *< 0.02, Figure 4) and with diaphragm active, cleaved calpain I protein levels for the LPS + EPA group substantially lower than the level obtained for the LPS alone group (*P *< 0.02, Figure 5).

## Discussion

### Respiratory muscle dysfunction in the intensive care unit

Severe respiratory muscle weakness is commonplace in patients requiring intensive care. This was best demonstrated by two recent studies that utilized magnetic stimulation techniques to objectively and non-volitionally assess diaphragm pressure generating capacity in critically ill patients requiring mechanical ventilation [23,24]. Both studies found that these patients had surprisingly severe diaphragm muscle weakness, with the patients in this study generating transdiaphragmatic pressures in response to magnetic twitch stimulation that averaged only 25% of the levels seen in normal healthy individuals. This severe respiratory muscle weakness compromises the ability of critically ill patients to breath, prolonging the need for mechanical ventilatory support in patients with lung disease and increasing the risk for recurrent respiratory failure once mechanical ventilation is removed [25].

Research has identified several pathophysiological processes that contribute to the development of respiratory muscle weakness in critically ill patients [1,2,26,27]. One important factor is infection, with several human studies demonstrating that infections can induce severe reductions in respiratory muscle strength [1,2]. Studies utilizing animal models of infection have confirmed this association, demonstrating the development of severe diaphragm weakness following endotoxin injection [28], after induction of pseudomonal pneumonia [29], following cecal ligation-puncture induced peritonitis [30] and after parasitic infections [31]. The mechanisms by which infections induce respiratory muscle dysfunction remain incompletely understood, but recent work has identified several cellular pathways that may contribute to the development of this problem. First, infections appear to increase diaphragmatic levels of oxygen derived free radical species (for example, peroxynitrite) and early administration of scavengers/inhibitors of reactive oxygen species have been shown to reduce the development of diaphragm dysfunction in several models of infection [32,33]. Second, work suggests that infections induce caspase activation in striated muscles (cardiac muscle, diaphragm, limb muscles) and that caspase mediated contractile protein cleavage may underlie, at least in part, some of the skeletal muscle and cardiac contractile dysfunction seen with infections [19,34]. Third, infections increase activation of the proteasome and calpain proteolytic enzyme systems which, in turn, are thought to contribute to skeletal muscle protein loss and atrophy during sustained infection [35-37].

### Effects of EPA

Several studies have shown that chemical inhibitors of several of the pathophysiological pathways identified in the preceding paragraph can significantly reduce respiratory muscle contractile dysfunction in animal models of infection. Agents that have been found to be effective in this manner include zVAD-fmk (a caspase 3 inhibitor), IEHD-fmk (a caspase 8 inhibitor), calpain inhibitor III (a calpain inhibitor), and PEG-SOD (a superoxide inhibitor) [32,34,38]. While all of these agents are reasonably effective in preventing the development of diaphragm weakness in animal models of sepsis, none of these agents are FDA approved to treat human disease, many have never been administered to humans, and the risks and side effects of most of these agents are virtually unknown.

In contrast, numerous studies have employed EPA in animals and humans and to date there are no reports of serious side effects. The current study suggests a potential new use for this agent and also identifies a previously unrecognized mechanism of action of the drug. We found that this agent largely preserved diaphragm strength following endotoxin administration. In comparison to previous studies examining endotoxin induced diaphragm dysfunction, the effects produced by EPA compare favorably to the beneficial effects produced by caspase inhibitors, p38 inhibitors and very selective potent calpain inhibitors (that is, calpain inhibitor III) in reducing endotoxin induced diaphragm dysfunction [28,32,34,39].

Another important finding of the current study is that the mechanism by which EPA appeared to prevent diaphragm dysfunction appears to be unrelated to previously published reports. Our findings indicate that this agent did not appear to inhibit caspase 3 activation, since caspase 3 activity assay levels were unchanged with this drug. While we cannot rule out an effect of EPA to modify free radical generation in selective muscle organelles (for example, lipid peroxidation of membranes), our data indicate it failed to substantially modify diaphragm protein carbonyl formation following endotoxin administration. The fact that this agent failed to inhibit caspase activation and failed to block protein carbonyl formation in the diaphragm also indicates that this agent did not act nonspecifically to downregulate the inflammatory response to endotoxin.

On the other hand, EPA did inhibit diaphragm calpain I activation, as evidenced by the fact this agent reduced both formation of active cleaved calpain I protein and reduced diaphragm calpain activity as gauged from a fluorogenic activity assay. This is the first report, of which we are aware, demonstrating that EPA has the capacity to inhibit calpain in an animal model of disease. There are two potential mechanisms by which calpain may have had this effect. First, calpain I is a calcium dependent enzyme whose activation is initiated by subtle increases in intracellular calcium concentrations in the vicinity of calpain molecules [38]. Once activated, this enzyme autocatalytically cleaves itself (see Figure 5, formation of a 78 kDa band in the diaphragm after endotoxin administration), with some data suggesting that this cleavage product is substantially more active at low calcium concentrations than the parent molecule [34]. Resting muscle calcium concentrations are known to increase in sepsis, and it has been postulated that this increase may be responsible for infection induced calpain activation [40]. EPA has been previously shown to incorporate into endoplasmic reticulum and sarcoplasmic reticulum membranes and thereby modulate calcium release and reuptake by these structures [41]. It is therefore theoretically possible that EPA may so alter sarcoplasmic reticulum (SR) function that it blocks cytosolic calcium increases and calpain activation following endotoxin administration. EPA has been reported to alter the activation of several kinases and calpain activation in muscle is thought to be, at least in part, modulated by kinase (that is, Extracellular Signal Regulated Kinase (ERK)) induced phosphorylation [42]. It is therefore also theoretically possible that EPA may have induced its effect by altering kinase mediated calpain I activation. Finally, one study has suggested that skeletal muscle calpain activation in sepsis is secondary, at least in part, to loss of activity of calpastatin, the endogenous calpain inhibitor [43]. It is therefore also possible that EPA administration may block calpastatin inactivation, thereby reducing calpain activity and calpain autocatalytic cleavage.

### Implications

As indicated earlier, EPA is thought to be a relatively innocuous agent with few side effects. In fact, this drug has been administered safely in studies of patients in critical care units with no reported morbidity or mortality attributed to the drug [44]. The current study suggests that EPA may be useful in critically ill patients to reduce infection induced diaphragm weakness. Moreover, since it appears that this agent acts by inhibiting calpain activation, it should also, theoretically, be effective in reducing diaphragm dysfunction in other situations in which calpain mediated diaphragm damage is thought to be present. Specifically, calpain activation has been implicated as the cause of mechanical ventilator induced diaphragm dysfunction in rats [26], and also, as a potential contributor to hyperglycemia induced diaphragm dysfunction (personal communication, LA Callahan). Future studies will be needed, therefore, to determine if EPA administration can be used therapeutically in the intensive care unit to prevent respiratory muscle weakness in infected, mechanically ventilated, and/or hyperglycemic patients. In addition, the protocol employed in the present study used EPA as a prophylactic agent, with initiation of administration of this drug at the time of endotoxin injection. Additional work will be needed to determine if this agent is also effective at latter time points and if it can rescue muscle function after an initial damaging insult.

## Conclusions

These data indicate that endotoxin-induced reductions in diaphragm specific force generation can be partially prevented by administration of EPA, a nontoxic immunomodulator that can be safely given to patients. We speculate that it may be possible to reduce infection-induced skeletal muscle weakness in critically ill patients by administration of EPA.

## Key messages

• Endotoxin-induced sepsis causes severe reductions in diaphragm strength.

• Administration of Eicosapentanoic acid (EPA) partially prevents endotoxin-induced diaphragm weakness.

• EPA appears to protect the diaphragm by inhibiting diaphragm calpain activation.

• We speculate that EPA may be useful in preventing weakness in critically ill patients.

## Abbreviations

Ac-DEVD-AMC: N-acetyl-Asp-Glu-Val-Asp-7-amino-4-methylcoumarin; AMC: 7-Amino-4-methylcoumarin; ANOVA: analysis of variance; CHAPS: 3-[(3-Cholamidopropyl)dimethylammonio]-1-propanesulfonate; DEVD-CHO: N-acetyl-Asp-Glu-Val-Asp-aldehyde; DNP: dinitrophenol; DTT: dithiothreitol; EDTA: ethylenediaminetetraacetic acid; EPA: eicosapentaenoic acid; ERK: Extracellular Signal-Regulated Kinase; FDA: Food and Drug Administration; HEPES: 4-(2-hydroxyethyl)-1-piperazine bethanesulfonic acid; HRP: horseradish peroxidase; IEHD-fmk: Ile-Glu-Thr-Asp-fluoromethylketone; LPS: lipopolysaccharide; NS: non significant; zVAD-fmk: carbobenzoxy-valyl-alanyl-aspartyl-[O-methyl]-fluoro-methylketone; PEG-SOD: polyethylene glycol superoxide dismutase; SDS: sodium dodecyl sulfate; SDS-PAGE: sodium dodecyl sulfate polyacrylamide gel electrophoresis; Suc-LLVY-AMC: Succinyl-Leu-Leu-Val-Tyr-7-amido-4-methyl-coumarin.

## Competing interests

The authors declare that they have no competing interests.

## Authors' contributions

GS wrote the final manuscript and performed the caspase and calpain assays. JV collected the force data. LAC contributed to the Western blotting. All authors reviewed the data and manuscript.
